# Why Loneliness Interventions Are Unsuccessful: A Call for Precision Health

**DOI:** 10.20900/agmr20200016

**Published:** 2020-06-17

**Authors:** Samia C. Akhter-Khan, Rhoda Au

**Affiliations:** 1 Department of Psychology, Humboldt University of Berlin, 10117 Berlin, Germany; 2 Department of Psychology & Neuroscience, Duke University Graduate School, NC 27705, USA; 3 Departments of Anatomy & Neurobiology and Neurology, Boston University Alzheimer’s Disease Center, Framingham Heart Study, Boston University School of Medicine, Boston, MA 02118, USA; 4 Department of Epidemiology, Boston University School of Public Health, Boston, MA 02118, USA

**Keywords:** isolation, social relationships, person-centered care, technology, intervention research, healthy ageing, dementia, cognitive decline, prevention, long-term care

## Abstract

**Background:**

Loneliness has drawn increasing attention over the past few decades due to rising recognition of its close connection with serious health issues, like dementia. Yet, researchers are failing to find solutions to alleviate the globally experienced burden of loneliness.

**Purpose:**

This review aims to shed light on possible reasons for why interventions have been ineffective. We suggest new directions for research on loneliness as it relates to precision health, emerging technologies, digital phenotyping, and machine learning.

**Results:**

Current loneliness interventions are unsuccessful due to (i) their inconsideration of loneliness as a heterogeneous construct and (ii) not being targeted at individuals’ needs and contexts. We propose a model for how loneliness interventions can move towards finding the right solution for the right person at the right time. Taking a precision health approach, we explore how transdisciplinary research can contribute to creating a more holistic picture of loneliness and shift interventions from treatment to prevention.

**Conclusions:**

We urge the field to rethink metrics to account for diverse intra-individual experiences and trajectories of loneliness. Big data sharing and evolving technologies that emphasize human connection raise hope for realizing our model of precision health applied to loneliness. There is an urgent need for precise, integrated, and theory-driven interventions that focus on individuals’ needs and the subjective burden of loneliness in the ageing context.

## INTRODUCTION

“*Now I see the mystery of your loneliness.*”—William Shakespeare

Loneliness may be a familiar feeling for most of us. Nevertheless, research has been rigorously trying for decades to better understand and study human loneliness from a scientific perspective. Most scholars have agreed on a definition of loneliness as a negative feeling that results from a discrepancy between desired and perceived social relationships [1]. Although this definition might represent the core of being lonely, we are still uncertain about how the experience of loneliness differs across people and contexts. Even less is known about how to successfully alleviate loneliness. Aside from the fact that loneliness is a generalized feeling that people across the world are suffering from, researchers have underestimated this important topic [2,3].

One example of underestimating loneliness, for instance, can be taken from the clinical setting. It is not an uncommon problem for older patients suffering from depression to be misdiagnosed with dementia [4]. Depression is closely linked to and often preceded by feelings of loneliness and may be a modifiable risk factor for dementia across the lifespan [5,6]. Combatting loneliness may therefore be a way to mitigate risk for cognitive impairment through inflammation and cortisol pathways (see [7] for review). Further, loneliness has been associated with multiple adverse health outcomes, including mortality (see [8] for review). A longitudinal study by Holt-Lunstad et al. [9] has estimated that loneliness increases the likelihood of death by 26%. Alleviating loneliness may therefore not only contribute to general human well-being and health but may also help reduce the economic costs of health care by decreasing the risk of morbidity, such as the skyrocketing amounts being invested in long-term care (LTC) and Alzheimer’s Disease (AD) [10,11].

Importantly, loneliness and social isolation are distinct phenomena [1]. Whereas social isolation refers to an objectively measurable degree of solitude [1,12], loneliness refers to a personal, subjective experience of one’s disappointment with current social relationships [1]. For instance, one may feel lonely even when one is surrounded by people. In fact, longitudinal data from the Amsterdam Study of the Elderly indicate that loneliness—but not social isolation—predicted the onset of dementia [13]. Our aim here is to review research on the subjective feeling of loneliness, not the objective measure of social isolation, and to highlight its complex relation to neurodegenerative diseases.

Despite increasing efforts to alleviate feelings of loneliness, researchers are still not close to solving this problem. That is, current interventions have not been found to be very effective [14–18]. We therefore aim to investigate the underlying reasons for why solutions have been ineffective, so as to shed light on misleading directions in current research on loneliness interventions. Moreover, we want to take the opportunity to learn from these issues and to generate new perspectives for the field of research on loneliness in the context of cognitive impairment. Taking a precision health approach, we investigate how research could come closer to revealing the mystery of loneliness and healthy ageing by making use of opportunities on the fronts of machine learning techniques and digital biomarkers. Ultimately, this review article contributes to new perspectives on the goal of ameliorating the global burdens of loneliness and dementia.

## PROBLEMATIZING: WHY ARE LONELINESS INTERVENTIONS UNSUCCESSFUL?

Scholars have investigated various interventions to tackle loneliness over the past four decades. Even though there has been increasingly more research on predictors and outcomes, we still have not found effective solutions to resolve, reduce, or prevent loneliness [14]. A comprehensive review evaluating direct and indirect interventions tackling loneliness for people with mental health problems concluded that no types of intervention have a robust evidence base [14]. Several reviews point out methodological considerations (e.g., study design, grouping heterogeneous approaches, focus on mode of delivery, clinical diversity) as key factors for unsuccessful results [15–19]. Although important, methodological issues do not seem sufficient to explain unsatisfying effect sizes and the ineffectiveness of interventions. For instance, technological interventions show diverging effects on different individuals, depending on *how* people make use of them [19]. Also, research has indicated that perceived control (i.e., a person’s belief about one’s own ability to manage the pursuit of one’s needs) may be a determining factor in the success of interventions [20]. Importantly, the interventions that seem to show more promise for ameliorating loneliness are those that consider adaptability to local contexts and include holistic community-development approaches [18,21]. Taken together, the lack of longitudinal, qualitatively strong studies evaluating the effectiveness of programs—and the small reductions in loneliness found to date—are consistent with the notion of improved but not recovered [15,22,23]. Understanding how complex loneliness actually is allows us to critically rethink current interventions and adjust them to real-life experiences of loneliness in the ageing context.

### Complexity of Loneliness

The complexity of loneliness is often overlooked in the research literature, with many interventions only addressing one specific element of loneliness [22]. For instance, interventions may target loneliness indirectly e.g., by addressing mental and physical health or living situation (macro level). Direct interventions, in contrast, may seek to target (i) the *quantity* of relationships by enhancing social skills and creating opportunities for increased contact to reduce social loneliness (meso level) or (ii) the *quality* of relationships by changing maladaptive cognition to reduce emotional loneliness (micro level). The recurrent question that seems to be common to various efforts is: What aspect of the vast and complex problem of loneliness can we address? Thus, a weakness of current approaches, which tend to focus on single interventions for the entire population (“one-size-fits-all”), is that such approaches ignore the heterogeneity of the construct and the diverse needs and contexts of the people who experience loneliness [14]. We need to go forward by taking a step back, looking at the big picture, and taking a holistic, person-centered view of the specific burdens that various individuals bear in their experiences of loneliness.

#### The loneliness-health cycle: An example

**Figure 1.**
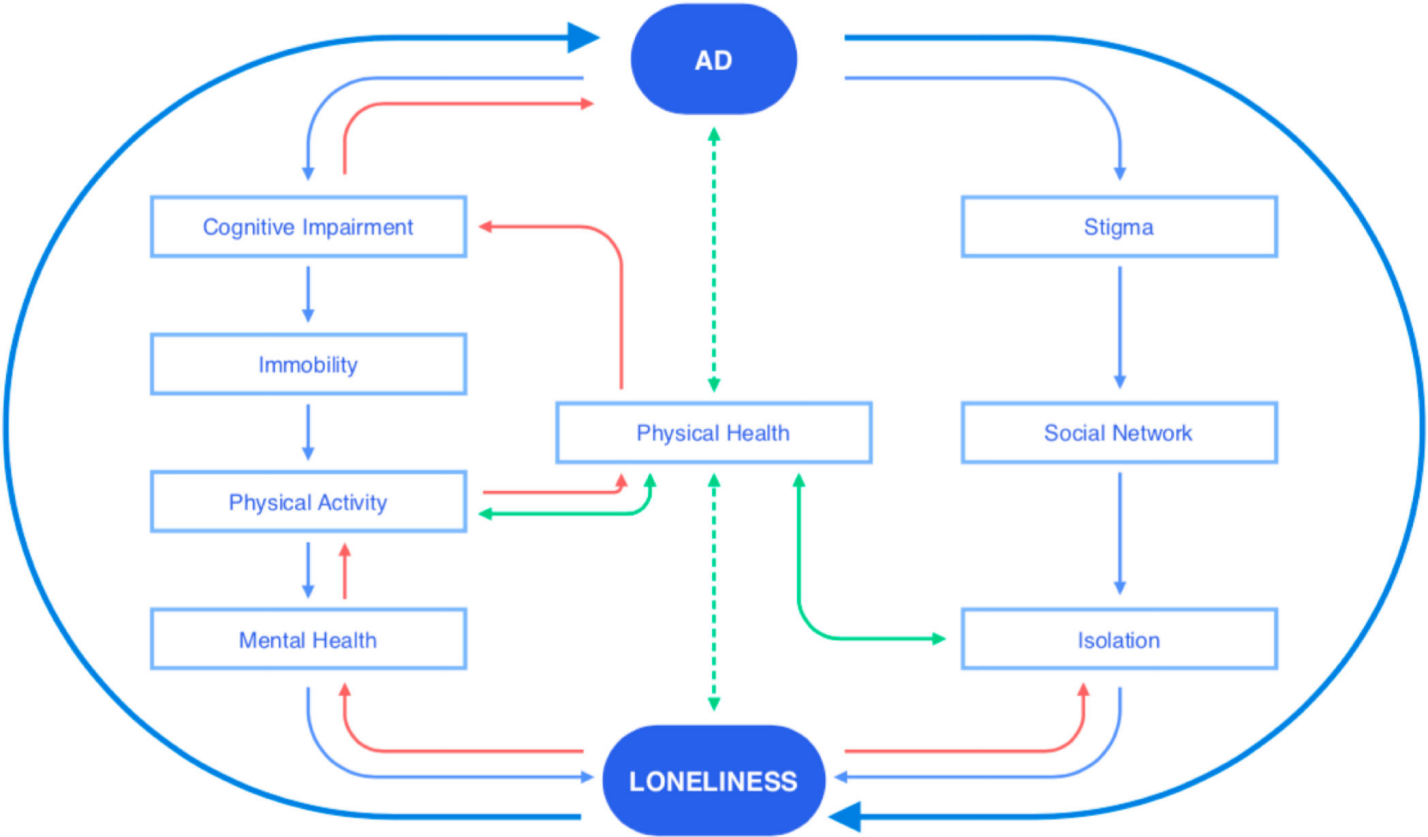
Possible interacting mechanisms of the loneliness-health cycle with the example of Alzheimer’s disease (AD). Bidirectional relationships between these constructs depict the complexity of how loneliness interacts with physical health. Light blue arrows indicate unidirectional pathways from AD to loneliness. Red arrows indicate unidirectional pathways from loneliness to AD, and green arrows indicate bidirectional pathways moderated by physical health and potential biomarkers.

To illustrate our following arguments, we give an example of the complexity and interacting pathways between loneliness and health. As mentioned in the introduction, loneliness is a strong predictor of health impairments and cognitive decline (see [5,8,24,25] for review). Several studies have shown that lonely people have different brain structures than non-lonely people, suggesting associations with biological risk-factors for cognitive decline and AD [26–28]. Yet, loneliness may also be an early manifestation of cognitive impairment, as indicated by higher beta amyloid burden, inflammation, and hypercortisolism in lonely, cognitively healthy adults [7,27]. As well, from the other direction, impaired physical and mental health (e.g., depression, anxiety, suicidal ideation, etc.) have been associated with a high risk of experiencing loneliness [7,14,29–31]. In Figure 1, we depict possible interacting pathways indicated by the literature for how an AD diagnosis could lead to feelings of loneliness, as well as vice versa. Some factors, such as increased stigma after a diagnosis, might specifically relate to the case of AD [32], as opposed to other health conditions, such as cardiovascular diseases.

This loneliness-health cycle is just one example of how intertwined the actual relationships between factors associated with loneliness are; loneliness is at the same time a predictor of certain variables and an outcome of other variables [7]. This example motivates questions such as: *Where* and *when* can we intervene—with *what* kind of interventions—and targeted to *whom*?

#### The heterogeneity of loneliness

Next to health (Figure 1), research has also uncovered multiple other predictors of loneliness (see [33] for review). However, only a few intervention studies discriminate between different sub-dimensions of loneliness, e.g., emotional loneliness versus social loneliness [22]. For instance, social loneliness, unlike emotional loneliness, was significantly more common in people with dementia [7]. Similarly, Cacioppo et al. [34] reviewed various theories on dimensions of loneliness, integrating them into a model encompassing intimate, relational, and collective types of loneliness. Whereas intimate loneliness was predicted by marital status, relational loneliness was predicted by frequency of contact with significant friends and family, and collective loneliness was predicted by the number of voluntary groups to which a person belonged [29,34,35]. Taken together, different predictors relate to different parts of the construct loneliness.

Further, the socio-cultural environment of an individual matters. For instance, the likelihood of feeling lonely may be exacerbated in the context of LTC settings or after being diagnosed with a neurodegenerative disease, reinforcing the cyclic feedback loops of AD and loneliness (see Figure 1) [7,15]. Other studies investigating loneliness indicate that day-to-day interactions vary between individuals, with some individuals meeting the same group of people every day and other individuals sustaining more heterogeneous relationships (e.g., [36]). Context also refers to differences between countries and cultures that continually shape human interactions and social relationships. For example, trends towards stronger individualism and urban migration may reduce opportunities for older people to provide and receive support within the community or intergenerational settings [3]. Accordingly, a cross-cultural study reported the need to tailor interventions to specific countries with regard to local health-related and economic resources [3]. As is the case for interventions for dementia [37], there seems to be a mismatch between the concept of loneliness in the research literature and the concept of loneliness in actual human experiences.

#### The person’s individual needs

Not accounting for different dimensions and contexts that shape loneliness leads us to the next major problem in current interventions: the lack of regard for individuals” varying situations and needs. Interestingly, individual needs are accounted for in the social needs perspective on loneliness that dates back to the early 70’s and 80’s [38,39]. These early theories suggested that distinct types of loneliness result from deficiencies in specific relationship provisions, i.e., positive contributions that humans bring into relationships. Attachment provisions, for instance, pertain to emotional loneliness (micro level) and needs for affection and opportunities for self-disclosure. In contrast, integrated involvement provisions pertain to social loneliness (meso level) and needs for respect and admiration. It may not be the lack of relationships in general but instead the lack of specific relationship provisions that causes loneliness [36]. For example, a person may have a wide social network of friends that fulfil integrated involvement provisions. However, this person was recently diagnosed with AD and now has a stronger need for attachment provisions, striving for emotional support by close family members and friends. A few years later, the context changes again as this person moves to a LTC facility where the needs may pertain more to integrated involvement provisions. Importantly, returning to the definition of loneliness, it is how individuals perceive their social relationships, not the objective state of their relationship, that is of ultimate significance here [1]. Thus, two people who have similar social relationships in the objective sense may feel different amounts of loneliness due to their different appraisals (see [36] for review).

How can we now relate this theory to intervention research given that people’s needs and subjective experiences differ? When we implement programs that target only social loneliness (e.g., a social skill intervention), we might only reduce loneliness in people whose integrated involvement provisions are not met, i.e., not in people in need for attachment provisions. Cacioppo et al. [34] argue that people are lonely for many other reasons that do not result from personal attributes, such as poor social skills. Thus, interventions teaching people to master social skills are only effective for people who are actually in need of social skill improvement [34]. While the social needs perspective only addresses one possible cause of loneliness, various theoretical understandings of how we approach loneliness influence the “active ingredients” of an intervention [17]. Accordingly, several scholars have highlighted the importance of theory-based interventions for their effectiveness [17,18,21]. Further, research indicates that we need more studies (specifically, RCTs) investigating how interventions apply to different groups of people that might have diverging needs [15,33,40,41], e.g., people with different kinds of cognitive impairments.

Next, it is essential to note that everyone will likely experience loneliness at some point in life [2]. Hence, the question that becomes relevant for intervention research is: When does loneliness become a burden? A number of studies suggest distinguishing between transient and persistent loneliness to account for the differences between natural, briefly occurring feelings of loneliness and more severe, chronic feelings of loneliness [20,40,42]. Another possible factor in the effectiveness of an intervention is the person’s motivation and openness to reduce loneliness [34,43]. For instance, an individual with AD might see loneliness as a significant personal burden but still not be motivated enough to meditate every day to try and alleviate this feeling because cognitive impairments make the required actions seem like an even greater burden.

In sum, the majority of intervention research focuses on testing one single intervention for all [22]. Although there are some newer studies and programs that acknowledge the complexity of loneliness and target their interventions to individuals’ needs and circumstances (e.g., [44,45]), the original model of “one-size-fits-all” research on loneliness interventions has not altered [14]. Due to the ineffectiveness of current interventions, we need to move towards acknowledging that interventions need to be tailored to individuals’ specific needs, especially in light of the complex effects of neurodegenerative diseases.

## NEW PERSPECTIVES

In 2019, the World Health Organization published a handbook calling for integrated care for older people (ICOPE) [46]. In line with this perspective, we want to propose new, transformational approaches focusing on person-centered and integrated care, that is, precision health. Unlike precision medicine, which treats symptomatic conditions with reactive measures, precision health detects presymptomatic patterns and implements proactive measures “just in time” [11]. Given the advances of new technologies and artificial intelligence, we are now able to shift approaches from treating diseases to ensuring wellness by prevention [11]. Digital measures, combining high-tech with high-touch (i.e., high focus on human interaction), coupled with standard assessments, may provide a more holistic and precise view of a person’s health in the real world outside of the clinic, revolutionizing our conception of healthcare [11].

### A Precision Health Approach to Loneliness

**Figure 2.**
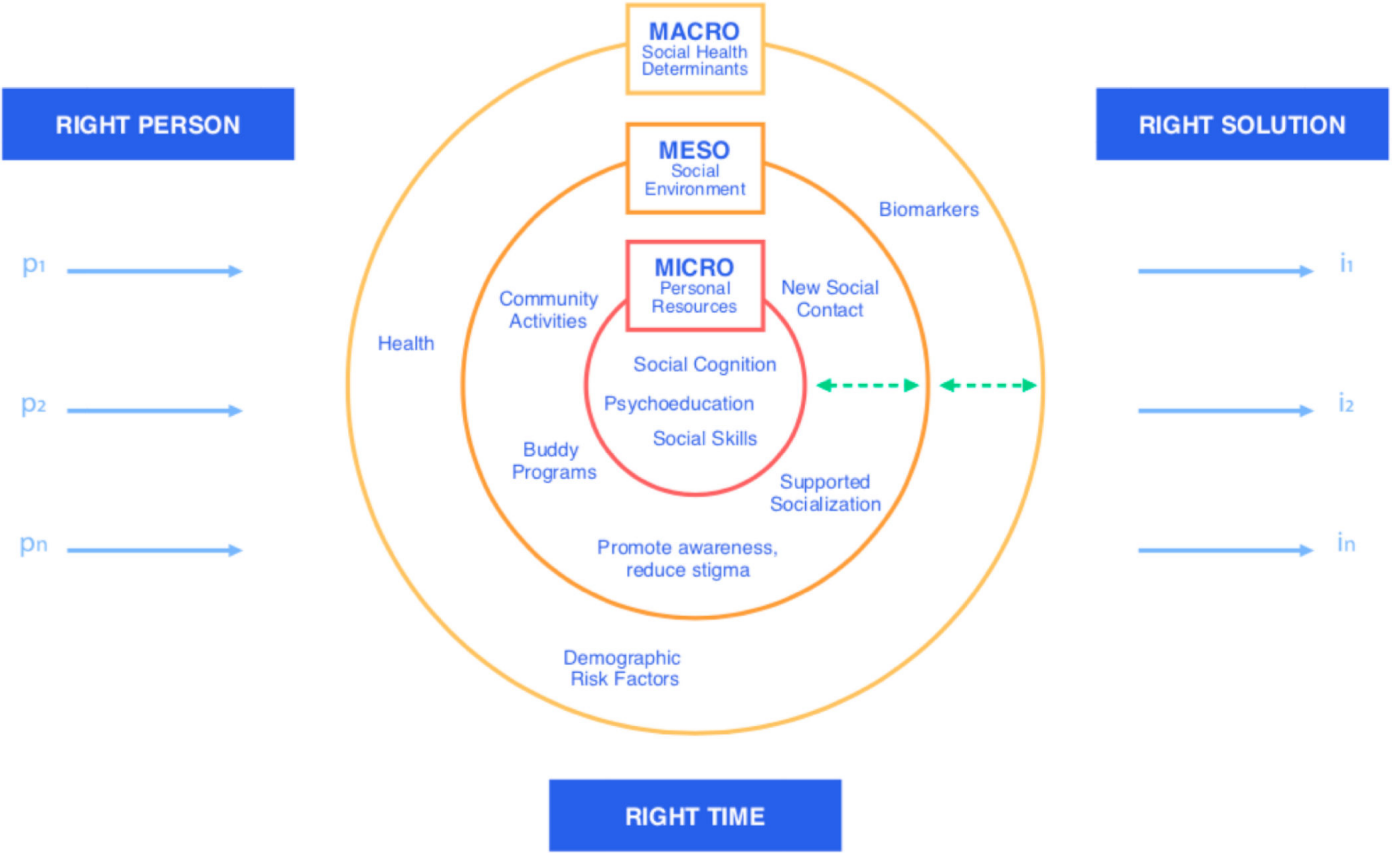
Precision Health Interventions for Loneliness. Interventions (i_1_, i_2_, i_*n*_) need to be tailored to individuals’ (p_1_, p_2_, p_*n*_) needs and situation. Individuals’ needs can relate to their personal resources (micro level), their social environment (meso level), and other interconnected factors, which are indirectly associated with loneliness (macro level). Indeed, interventions can be integrated to address needs on all three levels. Green arrows indicate that all levels influence each other, thus, are not distinct.

Returning to the question stated earlier of *how* we can change interventions, precision health can lead us one step closer to responding to people’s needs efficiently and accounting for diversity within individuals and loneliness itself. Here, we consider how healthy ageing approaches and loneliness interventions can be fruitfully informed by precision health (Figure 2).

#### The right solution

Although we previously shed light on the ineffectiveness of interventions, we point out that it is not necessary to invent new ones, as we already have various potential programs to alleviate loneliness. So, how can researchers implement existing interventions in order to make them more effective?

Given that loneliness is a complex construct with various dimensions, knowing various predictors for loneliness (e.g., relationship provisions), we can tackle emotional loneliness (micro level) through interventions that address cognition and appraisal on a personal level [22,34,47]. Social loneliness (meso level) may be addressed by interventions aimed at increasing social networks and connectedness through community activities or social media (e.g., [48]). On a macro level, we can implement programs that improve general health, e.g., treating hearing loss [49], and consider factors like living situation, immigration status, and socioeconomic status, which have all been shown to be social determinants of loneliness, well-being, and health [29,33,41,50].

Caring for human well-being involves biological, medical, psychological, physiological, and social perspectives, all of which are interconnected [30]. We need to integrate multiple interventions in order to treat a person holistically. Only a few studies have mentioned the need to investigate how interventions may be combined to achieve more effective outcomes, e.g., suggesting that programs for improving social skills and cognitions may work best in combination with other approaches [14,34]. The failure of current approaches, therefore, lies not in the interventions *per se*, but in the lack of integration and adjusting particular interventions to “the right person, at the right time” [11].

#### The right person

We previously highlighted how people experiencing cognitive decline might have different needs than cognitively unimpaired individuals (see Figure 1). Individuals differ inter-individually and inter-culturally not only in the occurrence of loneliness and dementia but also in the ways that they cope with neurodegenerative diseases and mental health (e.g., [2,51]).

This may become clearer when we consider a hypothetical example of a person with AD onset. Let us assume we take the person’s attachment needs into account and implement an intervention tackling emotional loneliness (micro level) by changing cognition because the person currently uses maladaptive cognitive coping techniques. Here, we would account for the person’s need, but we would still be taking a leap in the dark concerning which coping strategy the person would most likely benefit from. Rokach et al. [2,51] focused on the qualitative aspects of coping with loneliness and argued that there is not one unified strategy but in fact multiple effective coping techniques for loneliness. Results suggest that different choices among coping strategies (e.g., distancing and denial, religion and faith) are more prevalent within certain cultures and demographic groups due to their life experiences and the availability of such techniques [2]. Similarly, people with greater cognitive impairments might prefer and benefit from strategies that focus more on regulative coping (i.e., efforts to diminish emotional consequences of stress) than an active coping strategy that requires efforts to alter the troubled person-environment relationship [52]. How can we account for this diversity when designing interventions for loneliness in the contexts of heterogeneous ageing and dementia?

This challenge leads us to the issue of metrics, that is, the ways we measure loneliness, cognitive decline, and the effectiveness of interventions. The most common assessment tools for loneliness (see [29] for review) consistently manage to categorize lonely and non-lonely people across countries but overlook a substantial gap of variability in the intermediate categories, which makes it difficult to compare prevalence estimates between groups [29,31]. This variability and insensitivity between the extremes serve as an indicator for an even bigger problem with regard to what exactly we want to capture when measuring the subjective burden of loneliness. Current scales assessing loneliness do not account for the subjective burden of loneliness for the individual [31]. This may result from the consensus definition of loneliness that depicts it as a negative feeling, one that is implied to be equally negative for everyone [1]. However, it is possible, depending on the effectiveness of one’s own coping skills, that an individual reports feeling lonely often but nonetheless does not see loneliness as a burden. On the other hand, another person experiencing loneliness might appraise this feeling as a persisting encumbrance, which may reflect a maladaptive coping strategy [51]. Similarly, different individuals with AD may have different appraisals and states of awareness towards their cognitive impairments [5].

Hence, it is evident that we need new measurements that include assessments of subjective burden, motivation to change this state, as well as preferences for coping strategies. To better consider individuals’ contexts, we suggest that surveys for intervention research include demographic (e.g., marital status) and contextual data (e.g., psychological distress, religious and community engagement, cognitive impairment), which have each been shown to predict distinct dimensions of loneliness (e.g., [29,34]). Thus, we can account for risk-factors, as well as possible antecedents of loneliness, such as AD. Yet, the question remains open as to when, how long, and how often we implement these assessment tools to also account for loneliness as a transient, changing phenomenon across the lifespan [40].

#### The right time

When is the right time to intervene? From a lifespan perspective, research has shown us that coping strategies and preferences change throughout different developmental periods [40,52]. Likewise, different cognitive strategies may contribute to coping with loneliness at different phases of life [40]. Hence, the challenge expands to how we can account for not only inter-individual but also intra-individual and inter-temporal changes associated with loneliness.

**Figure 3.**
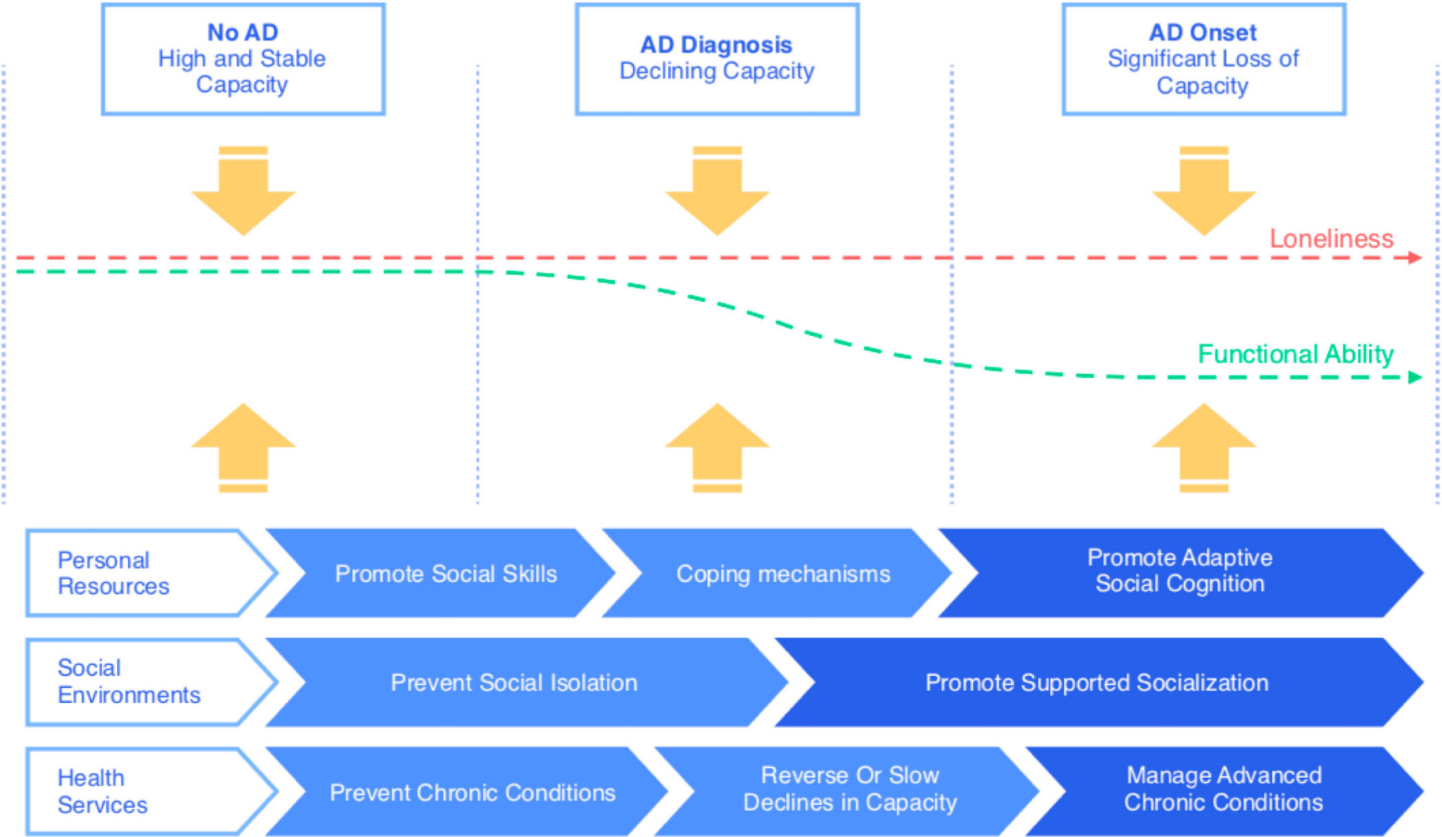
Interventions targeted at reducing loneliness across different time stages of AD onset. Loneliness can occur at any timepoint, whereas functional ability declines with AD onset. The three levels (see Figure 2) can address loneliness and functional ability differently across time. The primary focus lies on prevention through personal resources (micro), social environments (meso), and health services (macro).

A paradigm shift from treatment to prevention has taken place across various fields of healthcare (e.g., [5]). Due to increasing research on biomarkers of physical, cognitive, and mental diseases, we are now able to predict the onsets of a number of non-communicable diseases (e.g., [5,11,53,54]). The close association of mental and physical health with loneliness (see Figure 1) motivates our assumption that we can predict higher likelihoods of loneliness in people with these health impairments (e.g., [55]) and therefore intervene with a vast range of health promotion programs (reviewed in [41]). As illustrated in Figure 3, we have better chances to intervene before functional ability declines by implementing preventative interventions on micro, meso, and macro levels.

According to Perna et al. [54], making a prediction consists of two fundamental steps, which are applicable to loneliness. First, it is necessary to have the requisite information available on loneliness. This means gathering evidence about predictors and outcomes of loneliness, which various scholars have accumulated over the past decades (see [33] for review). Second, we need a model capable of generating predictions on the basis of this information, e.g., by linking individuals’ information to anticipated outcomes [54]. Relevant to this, machine learning (ML) procedures can develop predictive algorithms able to provide the most probable prediction on the basis of information for one individual person (supervised ML), or by identifying possible distinct subgroups from a population (unsupervised ML) [54]. Overall, the identification of relevant predictors remains the most important step in prediction and precedes the application of any algorithmic tool [54]. Knowing predictors and biomarkers of loneliness antecedents (macro level), e.g., for AD, provides a clear advantage [7]. Once the necessary tools and knowledge to predict loneliness are known, it is then possible to develop instruments of intervention at the intra-individual level.

Since loneliness can be experienced by anyone [51], in addition to identifying individuals at risk, it is important to engage in loneliness prevention from a broader, across-the-lifespan, perspective [40]. This includes raising early awareness to reduce stigma and promoting adaptive skills and coping strategies for healthy, satisfying relationships and attachment styles [14]. One effective way to raise awareness is education. For instance, the UK, driven by policies and campaigns to raise awareness for loneliness (e.g., [56]), will include a course on “Relationships Education” in primary and secondary schools’ curricula starting in September 2020 [57]. Lastly, there is a pressing need to combine government technical reports and other non-peer reviewed evidence on well-being with high quality research in order to to strengthen programs for preventing loneliness and cognitive decline [14].

To summarize, realizing precision health for loneliness presupposes a holistic approach to prevent loneliness across time on an inter-contextual, inter-individual, and intra-individual (inter-temporal) level. In the following section, we highlight the most important next steps for developing translational intervention research in line with our model.

#### Future directions

We have identified the need to:

- Rethink metrics to account for the heterogeneity and subjective burden of loneliness [29,31]
- Implement longitudinal and qualitative study designs to understand the trajectories of intra-individual coping mechanisms over time (e.g., [51,52])
- Find context-specific predictors for distinct dimensions of loneliness (e.g., [29])
- Investigate interventions targeted at specific sub-groups of people [31]
- Synthesize interdisciplinary data across stakeholders [14,54]
- Integrate interventions and develop complex evaluation methods (e.g., [58])
- Map interacting loops across levels of explanations for loneliness [59]
- Recognize loneliness as a generalized phenomenon that needs to be targeted beyond the borders of high-income countries [3,60]

Investigating how interventions could be integrated into holistic models for loneliness requires synthesizing data on two levels [59,61]. (i) We need to take a transdisciplinary approach combining data from different fields of research, e.g., psychology, data science, cognitive neuroscience, and social epidemiology [59]. (ii) Individuals’ phenotyping data, health risk factors, and data from continuous monitoring of subjective behavioral trajectories can be combined in longitudinal profiling methods (e.g., [61]). Finally, an essential part in precision health approaches is the implementation through technology [11]. Exploring the role of rapidly developing technologies can contribute further to answering the question of how we can create person-centered, integrated interventions to tackle loneliness and brain health effectively.

### The Role of Technology

Individualized treatment planning use time consuming and patient burdening clinical interviews and assessments with questionable cost-effectiveness and accessibility [42]. Technologies offer the means to precisely tailor interventions to individuals by collecting and generating real-time feedback integrated into people’s every-day life [11]. Using these tools, it may become realizable to support social connectedness as early as possible and buffer against the self-reinforcing relationship of loneliness and neurodegenerative diseases.

Technologies can help spread availability and access to interventions across country, race, and gender, and they can also facilitate the democratization of healthcare [11]. As such, smartphone applications, e.g., mHealth, can be implemented globally to tackle loneliness on all levels in an integrated manner (see Figure 2). On a micro level, emotional loneliness can be targeted through online psychoeducation, friendship enrichment, and social cognition programs (e.g., coping mechanisms, meditation) [62–64]. On a meso level, high-touch technologies, which connect people through digital communication interfaces, aim at increasing social connectedness (e.g., through promoting community activities of interest) [65]. These interventions can be specifically helpful for older people living apart from their children to reconnect and receive—as well as provide—emotional support [66–68]. More generally, on a macro level, technological interventions aiming to reduce loneliness can be linked with interventions and applications tackling social anxiety, depression, and physical activity to promote brain health (e.g., [63,69]). If we implement technologies as tools to prevent loneliness, we may be able to effectively intervene in the vicious cycle of AD and loneliness, even before the onset of cognitive decline (see Figure 1). Importantly, we are in need of an integrated technological ecosystem spanning both intervention and assessment to ensure a holistic approach to healthy ageing [11].

#### Towards measuring subjective experiences with objective measures

The *burden* of subjective experiences**—**cognitions, emotions, and behaviors**—**can be assessed and digitally monitored over time with remote recordings. Scholars have applied machine learning and data science techniques to find digital phenotypes of behavioral health, such as sleep or physical activity (macro level) [11]. Although the subjectivity of one”s experience (i.e., one’s personal burden) constitutes a core of mental health issues, remote recordings of physiological and behavioral data may also usefully predict mental health outcomes, such as stress, depression and bipolar disorder [70–73]. So far, only a few studies have investigated digital characterization of social isolation or loneliness (meso and micro levels) (e.g., [71,74]). The study by Doryab et al. [74] used passive sensing of mobile and fitbit data to detect low and high loneliness accurately. Even though this data could be used to get closer to predicting loneliness, it would still be unclear as to when and how to intervene. By measuring loneliness in a binary classification system, the granularity in subjective experience cannot be assessed, which is essential to distinguishing the burdens of transient versus persistent loneliness [20,31]. This leads us to the importance of considering the actual necessity and timing of intervention when assessing health by means of remote monitoring within the precision health framework (see Figure 3). Gideon et al. [75] have suggested a temporal normalization model to account for this issue, predicting clinical intra-individual mood changes from natural telephone speech data to determine the timing of an intervention.

Implementing assessments that only distinguish between lonely and non-lonely individuals as binary classification, leads us to the issue of measuring loneliness as a unitary construct and not accounting for distinct dimensions, e.g., emotional versus social loneliness [31]. Future research on technology and loneliness needs to incorporate measures that assess loneliness in a way that is appropriate to the actual experience in order to understand where the person’s relationship provisions are not met. Lastly, research should focus rather on intra-individual changes in loneliness trajectories, acknowledging heterogeneity [14,51], in order to precisely target integrated interventions to people at risk of cognitive impairments.

## CHALLENGES

When implementing technological interventions with older people, it is crucial to understand how technologies are being used, e.g., as a form of communication in high-touch technologies [21,68]. Scholars are investigating how interventions, e.g., for stress-reduction, can be matched to individuals’ preferences [70], and mathematical models can predict individual trajectories in mental states [54,76]. Nevertheless, technical illiteracy among older adults is a critical challenge that needs to be considered in the design of interventions [43]. Older people with dementia are in need of tailored technological interfaces that allow them to easily communicate with their loved ones despite cognitive impairments, while at the same time being aware of privacy implications [43]. This leads us to critical ethical questions for decisions on data sharing and privacy that need to be adequately addressed in the global context. These major challenges emphasize the need for various disciplines to work collaboratively [11]. Further, more research is needed on how to engage the full potential of technological devices in assisting efforts towards global mental health and well-being. This research may take the form of developing machine learning techniques to calibrate interventions for individual needs by implementing digital biomarkers in existing longitudinal datasets to track and predict intra-individual changes over time and, most importantly, in time. The unanswered question that is yet to be addressed is: Will we be able to predict changes in psychological well-being that are dependent on real-life human relationships?

When assessing loneliness through digital metrics we need to ensure that physiological and behavioral data collected from technological sources correspond to subjective reports of loneliness and identify diagnostic thresholds within individuals, not only across populations. The precision health approach requires not relying on technology as “the” singular solution [11]. Instead, an *ecosystem* of interventions introduced and realized through technologies can ultimately prioritize promoting and improving real-life human connections.

## CONCLUSIONS

The pressing need to find solutions to alleviate loneliness and promote healthy ageing has been increasingly acknowledged over the past decade. Yet, scholars and practitioners are underestimating the complexity of loneliness, specifically, its cyclic and exacerbating relationship with AD. As is the case for dementia, subjective burdens of loneliness vary across individuals, contexts (e.g., culture), and time. Thus, a “one-size-fits-all” approach is insufficient to treat loneliness in context. What we need is a better understanding of how different dimensions of loneliness can be tackled: on micro-, meso-, and macro-levels. We here introduced a precision health approach to loneliness that motivates new perspectives on mental, physical, and brain health to find the right solution, for the right person, at the right time. Realizing this approach will require various research efforts to further understand the heterogeneity of loneliness and develop machine learning techniques to identify prognostic digital biomarkers. These advances will soon allow us to predict changes of loneliness and brain health on an intra-individual level and target them with precise and timely interventions. Successfully reducing the burden of loneliness will ultimately drive down costs and obstacles that we are currently facing linked to AD and dementia. Despite various challenges lying ahead of us, we are confident that these new perspectives will move us towards a better understanding of human well-being and advance healthy ageing approaches globally.

## Data Availability

No data were generated from the study.

## ABBREVIATIONS

AD: Alzheimer’s Disease
ADL: activities of daily living
AI: artificial intelligence
ICT: information and communication technology
LTC: long-term care
ML: machine learning
NCDs: non-communicable diseases
RCT: randomized control trial
